# Muscle Protein Synthesis and Whole-Body Protein Turnover Responses to Ingesting Essential Amino Acids, Intact Protein, and Protein-Containing Mixed Meals with Considerations for Energy Deficit

**DOI:** 10.3390/nu12082457

**Published:** 2020-08-15

**Authors:** Jess A. Gwin, David D. Church, Robert R. Wolfe, Arny A. Ferrando, Stefan M. Pasiakos

**Affiliations:** 1 Military Nutrition Division, U.S. Army Research Institute of Environmental Medicine, Natick, MA 01760, USA; jessica.a.gwin.ctr@mail.mil; 2 Oak Ridge Institute for Science and Education, Oak Ridge, TN 37830, USA; 3 Department of Geriatrics, Donald W. Reynolds Institute on Aging, Center for Translational Research in Aging & Longevity, University of Arkansas for Medical Sciences, Little Rock, AR 72205, USA; DChurch@uams.edu (D.D.C); RWolfe2@uams.edu (R.R.W.); aferrando@uams.edu (A.A.F.)

**Keywords:** essential amino acids, protein, meal format, muscle protein synthesis, whole-body protein balance

## Abstract

Protein intake recommendations to optimally stimulate muscle protein synthesis (MPS) are derived from dose-response studies examining the stimulatory effects of isolated intact proteins (e.g., whey, egg) on MPS in healthy individuals during energy balance. Those recommendations may not be adequate during periods of physiological stress, specifically the catabolic stress induced by energy deficit. Providing supplemental intact protein (20–25 g whey protein, 0.25–0.3 g protein/kg per meal) during strenuous military operations that elicit severe energy deficit does not stimulate MPS-associated anabolic signaling or attenuate lean mass loss. This occurs likely because a greater proportion of the dietary amino acids consumed are targeted for energy-yielding pathways, whole-body protein synthesis, and other whole-body essential amino acid (EAA)-requiring processes than the proportion targeted for MPS. Protein feeding formats that provide sufficient energy to offset whole-body energy and protein-requiring demands during energy deficit and leverage EAA content, digestion, and absorption kinetics may optimize MPS under these conditions. Understanding the effects of protein feeding format-driven alterations in EAA availability and subsequent changes in MPS and whole-body protein turnover is required to design feeding strategies that mitigate the catabolic effects of energy deficit. In this manuscript, we review the effects, advantages, disadvantages, and knowledge gaps pertaining to supplemental free-form EAA, intact protein, and protein-containing mixed meal ingestion on MPS. We discuss the fundamental role of whole-body protein balance and highlight the importance of comprehensively assessing whole-body and muscle protein kinetics when evaluating the anabolic potential of varying protein feeding formats during energy deficit.

## 1. Introduction

The dose-dependent relationship between supplemental dietary protein and resting and post-resistance exercise skeletal muscle protein synthesis (MPS) is well described [1,2,3,4,5]. Moore et al. [4] reported that post-resistance exercise MPS demonstrates saturation kinetics and plateaus after ingesting a 20 g solution of whole-egg protein, as compared to 0, 5, 10, and 40 g in healthy young adults. Witard et al. [5] also demonstrated that, in healthy young adults, ingesting a 20 g solution of whey protein maximally stimulates resting and post-resistance exercise MPS, while ingesting 40 g increases protein oxidation and ureagenesis, without an additional anabolic stimulus. Similar work has been conducted in middle-aged adults [6], as well as older adults with anabolic resistance [7,8,9,10]. These well-designed dose-response studies clearly demonstrate that consuming 0.25–0.3 g and 0.4 g of protein/kg per dose maximally stimulates MPS in young and older adults, respectively [4,5,7]. As a result, these doses are promoted as effective post-exercise dietary strategies that optimize muscle adaptations to exercise when coupled with habitual protein intakes nearly twice the recommended dietary allowance [11,12,13]. Whether these recommendations derived from studies in healthy individuals during energy balance are adequate for individuals undergoing physiological stressors, including the catabolic stress of energy deficit, remains in question. Our work suggests these protein doses are not effective for mitigating lean mass (muscle) loss during military training that often results from energy deficit-mediated changes in MPS. We demonstrated no apparent stimulatory effect of feeding with 25 g of whey protein on MPS-associated anabolic signaling in individuals exposed to high altitudes (4300 m), before and after 22 days of acclimatization and severe energy deficit (40% energy deficit, −1766 kcal/day of energy balance) [14]. We also found no effect of providing four 20 g whey protein supplements per day on whole-body protein retention during a multi-day strenuous winter military training exercise that elicited a severe energy deficit (54% energy deficit, −3313 kcal/day of energy balance) [15]. These studies highlight the stressors and protein metabolism-related consequences routinely experienced by military personnel during strenuous training or combat operations, including high-energy flux, limited food availability, severe energy deficit, and, in some cases, environmental extremes. Establishing protein intake recommendations specific to scenarios that include energy deficit-induced catabolic stress may also benefit other healthy, primarily lean, populations that frequently perform under these conditions, such as weight-class sports athletes, ultra-endurance athletes, and wildland firefighters. 

Several factors contribute to energy deficit-induced lean mass loss, including downregulated MPS [16,17,18], blunted MPS-associated anabolic signaling responses to feeding [14], and increased whole-body protein oxidation [19,20,21]. The downregulation of postabsorptive and postprandial MPS is further exacerbated during severe energy deficit because dietary protein is used as a readily available, oxidizable energy source to meet increased whole-body energy demands [14,22]. While recommendations based on MPS responses provide valuable guidance for prioritizing muscle mass accretion and maintenance in healthy individuals during energy balance, they may overlook the potential benefit of protein intake on whole-body protein balance (whole-body protein synthesis—whole-body protein breakdown) during energy deficit. Feeding strategies that optimize the effects of protein on both whole-body protein balance and MPS are needed to mitigate or attenuate lean mass loss during sustained periods of moderate to severe energy deficit. 

Certain considerations should be acknowledged when identifying best practices to leverage the anabolic properties of dietary protein. First, the aforementioned dose-response studies, and a significant portion of the work on muscle protein turnover, only assess the effects of isolated intact proteins on MPS and do not evaluate other amino acid containing feeding formats. This is an important consideration, since peripheral essential amino acid (EAA) concentrations that directly stimulate MPS [23,24,25] are influenced by the feeding format. Therefore, eating varying formats such as supplemental free-form EAA, supplemental intact protein, or protein-containing mixed meals dictates postprandial peripheral EAA concentrations and MPS. Second, initial work suggests that the MPS saturation point following isolated intact protein feeding in healthy individuals is similar during energy balance and moderate energy deficit conditions. However, whether the MPS saturation point changes as the magnitude of energy deficit increases remains unknown [26]. Lastly, the studies used to inform current recommendations do not consider enhancing whole-body protein balance as a marker of optimal protein feeding amounts. An understanding of how feeding formats and related alterations in circulating EAA concentrations impact MPS and whole-body protein balance is required in order to expand the current recommendations and design feeding strategies for periods of energy-mediated catabolic stress. While several recent, well-written reviews [27,28,29,30] cover similar concepts, including protein quantity and quality, we sought to review and highlight specific format considerations and relate them to individuals undergoing energy deficit-induced stress. The objectives of this review are to summarize what is known regarding the effects of supplemental free-form EAA, supplemental intact protein, and protein-containing mixed meals on MPS during energy balance and present the importance of simultaneous measures of MPS and whole-body protein balance during energy deficit.

## 2. Considerations for the Application of MPS Measures

MPS is most commonly assessed using the precursor-product model, which involves the injection or infusion of amino acid tracers (precursor) and measuring their direct incorporation into muscle tissue (product) [31]. The fractional synthetic rate, or the rate of incorporation into the product, is calculated by dividing the rate of increase in product by the average precursor enrichment over the incorporation period, and is used to report acute changes (i.e., over several hours) in MPS [31]. It is critical to understand that MPS measures reflect only one aspect of muscle protein turnover and do not reflect protein anabolism without a concurrent measure of muscle protein breakdown. Ideally, muscle protein breakdown and MPS would be measured simultaneously to attain the net muscle protein balance. However, muscle protein breakdown measures present methodological challenges and accuracy concerns, especially during non-steady states (i.e., acute feeding and exercise) [32]. In addition, whether acute increases in MPS directly translate to long-term increases in muscle mass has also been questioned [33]. While some studies have demonstrated a good qualitative agreement between acute MPS measures and longer-term measures of hypertrophy-related outcomes [34,35,36], others have not [37,38]. The variance in agreement is attributed to the timing, frequency, and duration of measures, exercise type, training status, training protocol, and nutrition status (to include acute and habitual dietary protein/EAA intake). Beyond hypertrophy, MPS also serves to enable skeletal muscle adaptation to exercise through repair and remodeling, particularly after resistance exercise [39]. In this regard, MPS may be elevated as a response to exercise-induced muscle damage, but may not result in hypertrophy. While important, further discussion of these variables is beyond the scope of this review, and we direct the readers to other publications [33,40,41].

There has been a significant reliance on MPS measures alone to assess protein feeding responses due to the notion that MPS is more responsive and constitutes a greater contributing factor to muscle protein balance than muscle protein breakdown [32]. Although generally accurate during energy balance, this perspective may be problematic when translated to studies of severe energy deficit and other catabolic conditions. In some catabolic conditions, such as burn injuries, MPS is generally elevated due to increases in protein breakdown-derived intracellular amino acid availability [42]. Characterizing anabolism based solely on MPS in this scenario suggests that muscle protein is gained when it is actually lost due to greater elevations in breakdown than synthesis and the inefficient intracellular recycling of amino acids as a result of efflux and oxidation [43]. Although a valuable tool, there is also an inherent variability in MPS measures due to the stable-isotope methodologies employed (reviewed elsewhere [31,44,45,46]), study design, and magnitude of individual responses. These issues combine to increase the difficulty of direct comparisons from study to study and between laboratories [46]. Lastly, 40% of total-body protein is comprised of skeletal muscle, thus it is estimated that 33–50% of whole-body protein turnover is attributable to muscle tissue; however, the environmental context may alter the contribution [47]. Therefore, concurrent measures of MPS and whole-body protein turnover may provide a more holistic understanding of how feeding interventions impact body protein status.

## 3. Supplemental EAA and Protein Feeding Format Effects on MPS

**Figure 1:**
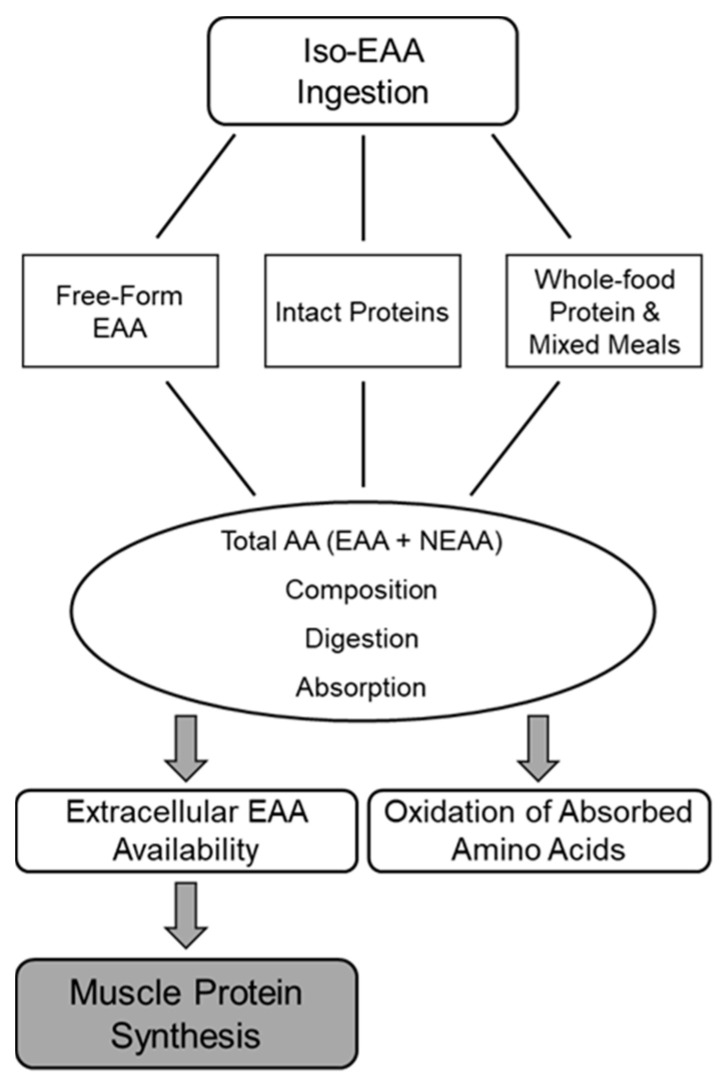
Factors moderating the effects of essential amino acids (EAA) on muscle protein synthesis.

**Figure 2:**
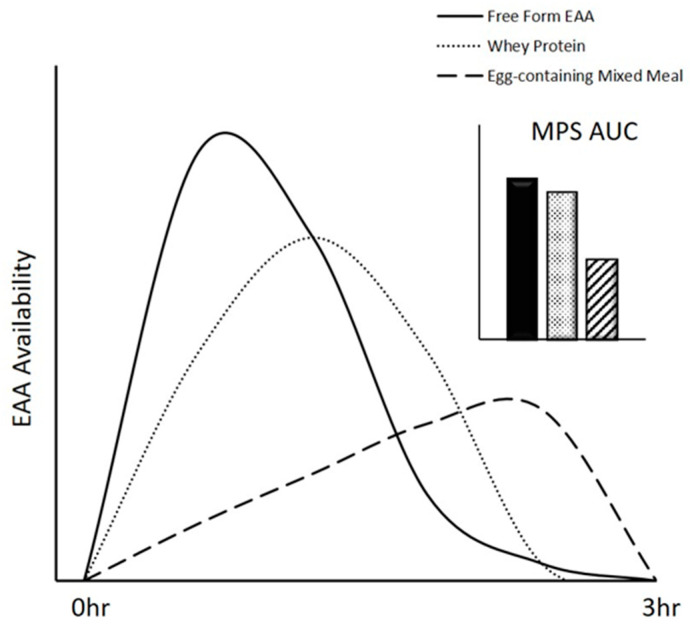
Theoretical comparison of magnitude changes in muscle protein synthesis (MPS) in response to the ingestion of supplemental free-form essential amino acids (EAA), whey protein, and a protein-containing mixed meal.

Amino acid feeding formats, which include supplemental free-form amino acid mixtures, supplemental isolated intact proteins (e.g., whey protein), whole-food proteins, and protein-containing mixed meals (Figure 1), differentially stimulate MPS. The varying effects of these formats on MPS are driven by the EAA content, digestion, and absorption kinetics of the particular format. These aspects, in turn, influence postprandial EAA concentrations, which are primarily responsible for stimulating MPS (Figure 2). Orally consumed free-form EAA do not require digestion, so they are absorbed and enter peripheral circulation rapidly, resulting in robust increases in EAA concentrations [48]. In contrast, isolated intact proteins, whole-food proteins, and protein-containing mixed meals must be digested before amino acid absorption. The co-ingestion of other nutrients may also influence their digestion and absorption, resulting in variations in postprandial EAA concentrations. Very few studies have directly compared the effects of supplemental free-form EAA, supplemental isolated intact proteins, whole-food proteins, or protein-containing mixed meals on postprandial EAA concentrations and MPS, though they have been independently studied in great detail. 

### 3.1. Total Amino Acid Content and Composition

**Figure 3:**
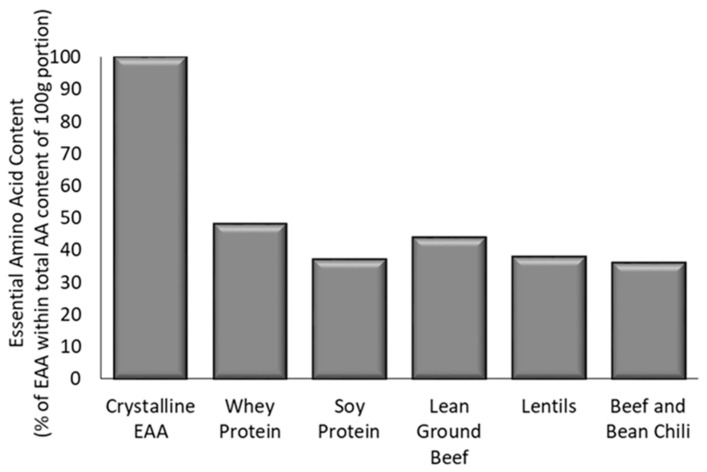
Relative essential amino acid content of free-form essential amino acids, various intact proteins, and a protein-containing mixed meal.

The quantity and composition of EAA varies considerably between supplemental free-form EAA, supplemental intact proteins, and protein-containing mixed meals (i.e., free-form EAA mixtures, dairy, plant, non-dairy animal proteins; Figure 3). This influences the degree to which MPS is stimulated, as there is a dose-dependent relationship between the EAA quantity ingested, postprandial EAA concentrations in circulation, and MPS [4,5]. For this reason, feeding formats that contain higher EAA quantities per volume (i.e., a higher EAA density) may be consumed in smaller amounts than formats with lower EAA quantities (i.e., a lower EAA density), while achieving similar postprandial increases in EAA concentrations and MPS [49]. Beyond absolute quantity, the differences in EAA composition also influence circulating EAA concentrations. 

Regarding isolated intact proteins, Tang et al. [50] used a unilateral leg-resistance exercise model to compare resting and post-exercise MPS after ingesting 22 g of whey, casein, and soy protein. The treatments were iso-EAA (10 g), but differed in EAA composition (whey: 2.3 g leucine; casein: 1.8 g leucine; soy: 1.8 g leucine). The EAA concentrations 30 min post-ingestion were 80% higher than the postabsorptive concentrations after ingesting whey protein. The postprandial increase after ingesting whey was greater than that of soy (30%) and casein (16%), which were the same. After 60 min, the postprandial EAA concentrations continued to increase, and were greatest after ingesting whey (42%), followed by soy (30%), and then casein (14%). Resting MPS measured 3 h post-ingestion was the same after ingesting whey and soy, but both were greater than casein. However, post-exercise MPS was greatest after ingesting whey, followed by soy and then casein. These findings highlight the interactive effects of EAA composition, digestion, and absorption kinetics on MPS across different protein sources within a given protein/EAA format (i.e., isolated intact protein) that provides comparable amounts of EAA.

Blending isolated intact proteins may be one method to optimize MPS by leveraging variations in protein digestion and absorption kinetics. Reidy et al. [51] compared the effects of consuming whey (18 g protein; ~9 g EAA, ~1.9 g leucine) or an iso-nitrogenous soy-whey-casein blend (19 g protein; ~9 g EAA, ~1.8 g leucine) 1 h following exercise completion on post-exercise MPS. Whey protein elicited higher and earlier peak postprandial EAA concentrations, whereas the blended protein led to a delayed, gradual, and sustained increase in the EAA concentrations. Despite these differences, both the treatments produced a >50% increase in the EAA concentrations over the 5 h post-exercise period. The MPS rates mimicked the EAA concentrations, such that MPS was the same between treatments across the entire 5 h period. Interestingly, the MPS rates in the latter 2–4 h period were only increased above rest by the blended protein. These data suggest that there are temporal differences in the postprandial EAA kinetics and MPS responses between single-source, isolated intact proteins and blended isolated intact proteins. The same laboratory conducted a follow-up study [52] in older men. While the total protein quantity was increased (30 g protein), the findings were the same, such that the increases in EAA concentrations and MPS were similarly elevated following whey or protein blend ingestion. Overall, these data support that similar increases in postprandial EAA concentrations stimulate and sustain MPS to a similar extent. These data may also support the co-ingestion of whole-food proteins (i.e., milk and eggs, beef and beans) as a practical way to achieve optimal EAA intakes. 

The composition of amino acids provided in supplemental free-form EAA mixtures may vary to the same extent as (or more so than) the EAA compositions in intact proteins. The wide variation is almost certainly the result of intentions to develop the best combination of free-form amino acids to support MPS. For example, Glynn et al. [53] sought to determine whether manipulating the leucine content (1.8 (i.e., consistent with the proportion in whey protein) vs. 3.5 g leucine) within 10 g of free-form EAA had any greater benefit for stimulating resting MPS in young adults. The EAA mixtures produced similar postprandial EAA concentrations, which was in agreement with similar changes in MPS, although MPS was stimulated to a greater extent at 60 min by the mixture with the higher leucine content. These findings suggest that 10 g of EAA with a leucine content of 1.8 g is sufficient to achieve a maximal MPS response in young healthy adults at rest.

In addition, increasing the total EAA content of a feeding format by enrichment with one or more additional free-form EAA may augment MPS. Adding EAA to a low dose of whey protein (10 g) or to a protein source with lower EAA content (soy protein relative to whey protein [54]) may stimulate MPS to the same extent as a standard dose of whey protein (20 g). The enrichment of low-dose intact protein ensures an adequate amount of EAA to increase the EAA concentrations required to stimulate and sustain MPS. Churchward-Venne et al. [55] examined the effects of consuming low-dose, leucine-enriched whey (8.4 g protein, 5.14 g EAA, 3 g total leucine), low-dose, EAA-enriched whey (12.55 g protein, 9.29 g EAA, 0.75 g total leucine), or 25 g of whey (25 g protein, 11.54 g EAA, 3 g total leucine) on resting and post-resistance exercise MPS. Postprandial EAA concentrations followed similar patterns for the leucine-enriched whey and the EAA-enriched whey, which increased to peak concentrations by 1 h (both greater than ~75% above basal). In contrast, the 25 g whey led to a greater EAA peak (greater than ~90% above basal) from 1.3–2 h versus the other two groups. Resting and post-exercise 3 h MPS increased similarly across all groups. However, post-exercise 3–5 h MPS remained elevated following 25 g whey, but not in the other groups, regardless of condition. The greater post-exercise 3–5 h MPS in the 25 g whey group may be attributed to the total circulating EAA concentrations, 30 min delay in the peak concentrations, and total amino acid quantity ingested. This study supports several key concepts. First, stimulating MPS over a 1–3 h postprandial period may be achieved by enriching lower quantities of isolated intact proteins with EAA, such that postprandial EAA concentrations are at least 50% greater than postabsorptive concentrations. Second, it seems that more total protein supports a sustained stimulation of MPS, particularly after exercise.

### 3.2. Digestion and Absorption

The protein digestion and absorption rates of intact proteins encompass the rate at which proteins are catabolized to peptides and free amino acids and the partitioning of these amino acids between the splanchnic and peripheral tissues [56]. Proteins can be broadly classified as fast (i.e., rapid digestion and absorption) or slow (i.e., prolonged digestion and absorption) based upon the peripheral EAA appearance, regardless of the amino acid composition [56]. Therefore, ingesting either fast or slow intact proteins or manipulating the timing of their ingestion may be methods to optimize postprandial EAA concentrations and MPS. One approach to examine the impact of digestion and absorption on postprandial EAA concentrations and subsequent MPS, while controlling for differences between amino acid compositions, is to administer the same quantity of protein in varying portions at differing time points. Areta et al. [57] examined the effects of consuming 80 g of whey (~38 g EAA) as two large boluses (i.e., 40 g whey, ~19 g EAA), four intermediate pulses (i.e., 20 g whey, ~10 g EAA), or eight small pulses (i.e., 10 g whey, ~5 g EAA) across 12 h on EAA concentrations and MPS. The EAA concentrations in the first 6 h of recovery (~19 g of EAA delivered in all treatments) did not differ from the baseline after the intermediate and small pulse treatments. However, the bolus treatment increased EAA concentrations by 135%. During the final 6 h of recovery, EAA concentrations increased by greater than 50% across the treatments. Overall, the bolus treatment lead to a greater peak in EAA concentrations (~140%) compared to the intermittent (~66%) and small pulse treatments (~80%). The intermediate treatment only increased the EAA concentrations from 6–7 h. In contrast, the delayed increases in EAA concentrations following the small-pulse treatment were sustained throughout the 12 h. Regardless of the differences in circulating EAA, 4 h MPS increased similarly across treatments. However, between 4–6 h and 6–12 h, MPS was greater following the intermediate versus the small pulse and bolus treatments. The differences between the EAA concentrations and MPS in this study are in line with the concepts of an EAA concentration threshold and saturation point for stimulating MPS, such that any further provision of EAA has no further stimulatory effect [58,59]. These data suggest that the EAA concentrations following the small pulses did not reach the threshold to maximally stimulate MPS, and that the EAA quantities in the bolus perhaps saturated the MPS stimulatory mechanism. These findings support that feeding formats which augment MPS under energy balance conditions are those that provide adequate EAA (i.e., 20 g whey, ~10 g EAA) and have fast to moderate digestion and absorption kinetics.

The digestion and absorption kinetics of whole-food protein ingestion are also altered by the food form (solid versus liquid) and food matrix (non-amino acid components). A study by Burd et al. [60] examined the post-resistance exercise effects of beef versus milk (30 g protein and 13 g EAA) on MPS. Circulating phenylalanine concentrations were greater following beef versus milk from 1–2.5 h, while leucine concentrations rapidly increased after the milk (40%) and were greater than the beef (10%) 30 min post-ingestion. Yet, the beef led to greater peak and overall leucine concentrations (> 100%) from 1.5–2 h. The 2 h MPS was higher following the milk compared to the beef, whereas 5 h MPS was equal between foods. These data indicate that temporal differences in circulating EAA concentrations and MPS exist between whole-food proteins. 

## 4. The Importance of Measuring MPS and Whole-Body Protein Turnover Together

The determination of optimal protein feeding strategies have been largely based on conventional myo-centric studies that only quantify MPS. This approach is suitable when the primary goal of protein feeding is to support muscle-specific adaptations. However, the use of MPS for evaluating an optimal feeding response fails to account for any further benefit on whole-body protein balance once MPS is saturated. EAA that remain in circulation after MPS is maximally stimulated may still be used to stimulate protein synthesis elsewhere in the body and reduce the reliance on precursor amino acids derived from energy deficit-induced upregulations in protein breakdown to support synthesis. The combination of increased whole-body protein synthesis and/or decreased whole-body protein breakdown is greater whole-body protein balance. Recent work from our laboratory highlights the value of simultaneous MPS and whole-body protein balance measures (62). Moreover, examining both muscle and whole-body measures is particularly important when developing recommendations for protein intake during catabolic stress, because MPS is not a priority amino acid-requiring process under these conditions. In contrast, life-sustaining processes, including acute-phase protein synthesis, immune function, wound healing, and energy production, are prioritized and upregulated during catabolic stress. Since skeletal muscle is the only labile amino acid source, an efflux of amino acids will be directed towards these important processes during this condition. There is also a greater dependence on whole-body protein breakdown to supply the amino acid precursors needed to sustain these processes [42,61]. Thus, an attenuation of exacerbated protein breakdown is a principal goal of protein intake during catabolic stress. 

Although several methodologies may be used to assess whole-body protein balance, our work compares the feeding effects of protein and free-form EAA feeding formats using the continuous infusion of EAA tracers. For a more detailed discussion of this method and others, we direct the readers to recent reviews [43,62]. The utility of whole-body protein turnover measures may be considered limited by the inability to determine the effects of feeding or training interventions on specific tissues. While technically correct, whole-body protein turnover measures do provide a holistic assessment of systemic changes in multiple body protein pools (i.e., skeletal muscle, organ, and splanchnic proteins), which is useful when assessing whether nutrition interventions meet total-body protein requirements. Perhaps most importantly, food is consumed at the whole-body level, and all nutritional guidelines have been based on whole-body responses (mainly nitrogen balance). Whole-body protein kinetics can be determined more rapidly than nitrogen balance, and can be performed using an experimental design that allows individuals to be used as their own control.

As previously stated, skeletal muscle contributes 40% of total-body protein and comprises ~33–50% of whole-body turnover measures [47]. It is important to consider how fluctuations in MPS are related to whole-body protein turnover and whether this relationship is moderated by exercise type. This is because the type and intensity of exercise stimulus dictates the MPS and hypertrophic response [63], and therefore may change the contribution of MPS to changes in whole-body protein turnover. To our knowledge, no studies have directly compared the effects of exercise type on the relationship between MPS and whole-body protein turnover. However, Koopman et al. [64], reported a correlation between post-resistance exercise MPS and whole-body protein balance following the ingestion of carbohydrate; carbohydrate and whey; or carbohydrate, whey, and leucine. Other work [8] from this laboratory also demonstrates a general agreement between the dose-dependent increases in post-resistance exercise MPS and increases in whole-body protein balance following protein ingestion. There also seems to be agreement between increases in MPS and increases in whole-body protein synthesis following endurance exercise [65]. Further investigation directly comparing this relationship is warranted and should emphasize a number of contributing factors, including training status, intensity, duration, age, and nutritional status.

The same format-related considerations for achieving the optimal quantity, composition, digestion, and absorption of protein and/or EAA are relevant for optimizing whole-body protein balance. For example, one of our studies [66] showed that consuming a mixed meal providing 70 g protein (~32 g EAA) results in greater whole-body protein balance compared to one providing 40 g of protein (~18 g EAA), despite comparable MPS rates. The enhanced whole-body protein balance was attributed to both the suppression of protein breakdown and the stimulation of protein synthesis. In addition to the quantity of protein/EAA, the composition of EAA within a meal format also has implications for enhancing whole-body protein balance. We recently demonstrated [67] the effects of consuming iso-nitrogenous, iso-caloric egg-based (26 g protein, ~10 g EAA), or cereal-based (26 g protein, ~7 EAA) mixed meals on whole-body protein turnover and MPS. Importantly, while iso-nitrogenous and iso-caloric, this was a comparison of whole-foods within formats typically consumed in the “real world”. EAA concentrations were greater following the egg-based meal versus the cereal-based meal. Whole-body protein balance was also greater following the egg-based meal compared to the cereal-based meal and primarily due to a greater suppression of protein breakdown. MPS did not differ between the formats. These data indicate that protein-containing mixed meals with differing EAA compositions are capable of eliciting differences in whole-body protein balance, despite delivering a similar protein quantity as well as comparable MPS responses. Lastly, EAA-enriched intact protein formats may be a promising approach for enhancing whole-body protein balance and provide benefits beyond achieving optimal MPS. Our laboratory compared whole-body protein turnover and MPS following ingestion of low-dose EAA-enriched whey (5.6 g protein, 3.2g free-form EAA), high-dose EAA-enriched whey (11.2 g protein, 6.4 g free-form EAA) and a commercially available whey-based supplement (12.6 g protein, 2.4 g EAA) [68]. Whole-body protein balance was greater following the high-dose EAA-enriched whey versus the other treatments due to a greater suppression of protein breakdown. Only the high-dose EAA-enriched whey increased MPS above basal values. While the increased MPS response is in agreement with the greater whole-body protein balance, the magnitude of these responses indicated the treatment led to greater benefit at the whole-body level. That is, if only the MPS response was evaluated to determine the efficacy of the high-dose EAA-enriched whey versus the other treatments, then it would appear rather modest. Whereas, when the MPS and whole-body protein balance responses are examined simultaneously, there is a robust feeding effect, clearly indicating the benefit of the high-dose EAA-enriched whey in supporting protein kinetics compared to the other treatments. Together, these data support the inclusion of simultaneous MPS and whole-body protein balance measures to assess the efficacy of feeding formats to support total-body protein status and that varying formats moderate feeding effects. 

## 5. Maximizing MPS and Whole-Body Protein Status during Energy Deficit

Negative energy balance generally results in muscle mass loss, the extent of which corresponds to the degree and duration of the deficit incurred [69,70]. This relationship is concerning for healthy, non-obese individuals who have a greater proportion of body mass derived from fat-free mass. Military personnel are one example of an at-risk population that undergoes frequent and repeated exposures to energy deficits in both training and operational environments. Nutritional countermeasures to attenuate muscle mass loss and subsequent decrements in health and physical performance are crucial. There is general consensus that providing more than the recommended dietary allowance for protein (0.8 g/kg body mass/day) can counteract the detrimental effects of energy deficit on lean mass. Controlled laboratory studies consistently show that increasing dietary protein intake (1.6–2.4 g/kg body mass/day) during moderate energy deficit (≤ 40% total daily energy requirements) prevents lean mass loss by restoring MPS rates to those observed during energy balance and maintaining postprandial anabolic sensitivity to high-quality, protein-containing meals [26]. As mentioned, our recent work [15,71] demonstrates that a single dose of whey protein within the recommended amount, 20–25 g, is inadequate to support muscle mass during conditions when energy deficit is severe. The apparent diverging effects of higher protein feeding during moderate and severe energy deficit suggest that the magnitude of energy deficit is an important moderator of whether dietary protein is used to support protein synthesis and spare lean mass, or whether it is diverted towards oxidative energy metabolism. 

There are limited studies examining MPS following acute supplemental free-form EAA or intact protein feeding in healthy populations exposed to energy deficit. The studies that have been conducted in our laboratory [16,72,73], and others [17,18,74,75,76] have largely focused on the interactions of energy deficit (i.e., typical weight loss or unavoidable, moderate to severe energy deficit) and supplemental free-form EAA or intact protein feeding on MPS. To our knowledge, only two have assessed acute MPS in response to varying quantities of supplemental free-form EAA or intact protein during moderate energy deficit [18,72]. Areta et al. [18] examined post-resistance exercise MPS after ingestion of 15 g (~7.5 g EAA) and 30 g (~15 g EAA) of whey protein. There was a dose-dependent increase in MPS; however, this increase only equated to the postabsorptive rates commonly observed during energy balance. Since quantities greater than 30 g of whey protein (15 g of EAA) were not included, the maximal stimulation of MPS could not be determined. Recent work [72] from our laboratory demonstrated that post-resistance exercise MPS was not further stimulated by ingesting 24 g (0.3 g/kg body weight) of free-form EAA when compared to 8 g (0.1 g/kg body weight). The discrepancies in MPS response between these two studies may be related to differences between the effects of whey protein and free-form EAA on MPS. For example, Areta et al. provided an intact protein which delivers both essential and non-essential amino acids and requires digestion before absorption, whereas we only provided rapidly absorbable free-form EAA. However, our findings coincide with a similar dose-response study examining free-form EAA [25] conducted under energy balance conditions. These studies suggest 8–10 g of EAA [25,72] optimally stimulate MPS regardless of the energy balance and moderate energy deficit conditions. However, further study of these doses provided in other feeding formats (i.e., plant-based intact proteins or whole-food proteins in mixed meals) or under conditions of greater energy deficit severity (i.e., >30% energy deficit) is warranted. 

In addition to MPS measures, our recent work [72] also included whole-body protein turnover measures. Despite no difference in MPS stimulation, the 24 g EAA dose increased whole-body protein synthesis and attenuated whole-body protein breakdown, which resulted in enhanced whole-body protein balance compared to the 8 g EAA dose. The enhanced whole-body protein balance following the 24 g EAA dose was consistent with greater postprandial circulating EAA concentrations compared to the standard dose. In this regard, there was no further benefit of greater circulating EAA concentrations for stimulating MPS. However, the elevations in circulating EAA provided precursor amino acids to meet whole-body protein synthesis requirements, which likely contributed to a reduction in the reliance on whole-body protein breakdown to supply precursor amino acids [77,78]. The effect on whole-body protein balance is particularly significant when considering the importance of preventing or attenuating extensive whole-body protein breakdown (i.e., beyond the breakdown required for normal tissue maintenance and repair) and related body protein loss during energy deficit [79]. As stated, muscle is not the priority tissue during catabolic stress. Instead, the body relies on muscle as a readily available supply of precursor amino acids used to meet the increased amino acid requirements for life-sustaining processes or to be oxidized as fuel to meet increased energy demands. Reliance on muscle protein breakdown may be increased further if amino acid precursors sourced from the diet are limited due to inadequate amount and/or quality of protein or EAA intake.

## 6. Knowledge Gaps Surrounding Protein and EAA Formats to Support Muscle Protein Synthesis and Whole-Body Protein Balance under Energy Deficit

The advantages and disadvantages of supplemental free-form EAA, supplemental intact protein, or protein-containing mixed meals depend on the populations and conditions in which they are consumed. Optimizing the feeding format-related effects on MPS, whole-body protein balance, and muscle mass may be less concerning for healthy individuals adhering to daily food intake patterns that meet optimal protein intake recommendations and daily energy requirements. Yet, feeding formats may have large implications for delivering the quantity and quality of protein and/or EAA, as well as total energy needed to support MPS and total body protein for individuals during energy deficit. Feeding strategies under these conditions must leverage formats that deliver optimal amino acid quantity and composition within a highly digestible and absorbable food format. As stated, military personnel are one example of an otherwise healthy population consistently exposed to multiple catabolic stressors including energy deficit [79]. Military personnel generally subsist on combat food rations during strenuous, sustained trainings and operations that elicit moderate to severe energy deficits. Current rations, while designed to provide adequate nutrition, are rarely consumed as intended due to logistical constraints and consumer acceptability (i.e., reduced appetite, high desire to carry fewer food items to reduce weight) [80]. In addition, the majority of protein-containing ration items contain protein sources with suboptimal EAA compositions and are primarily components of mixed-macronutrient meals, which may not optimally stimulate and sustain MPS or whole-body protein balance. While the current evidence provides initial direction for developing more optimal feeding formats, there are several knowledge gaps. First, although there appears to be supporting evidence for the efficacy of enriching intact proteins, further work is required to define the exact dosages and amino acid compositions that will deliver the greatest benefit. Similarly, the comparable effects of blended and single-source isolated intact proteins on MPS support blending these protein sources. However, whether this relationship remains equivalent between single-sources and blends of whole-foods (i.e., whole-food co-ingestion) must be tested. Overall, the information surrounding whole-food proteins is limited [28,29]. Further characterization of whole-food proteins is required and may inform the development of meal patterns specifically aimed at achieving optimal protein and/or EAA intake. Lastly, for scenarios of energy deficit, additional studies are needed to determine whether feeding formats that provide greater quantities of amino acids and/or total energy beyond the current recommended dosages are more advantageous compared to those that do not. Greater quantities of both essential and nonessential amino acids and/or energy may be required during energy deficit to offset or attenuate increased catabolism and support increased whole-body energy and protein requirements.

## 7. Conclusions

In this review, we summarized how supplemental free-form EAA, supplemental intact protein, and protein-containing mixed meal formats moderate MPS during energy balance. It appears that ingesting formats that provide ~20 to 30 g or 0.25–0.30 g/kg protein or ~10g or 0.10g/kg free-form EAA per dose optimizes MPS in healthy, non-obese individuals consuming habitual protein intakes of 1.6 g/kg/day during optimal conditions, such as following resistance exercise and in the absence of energy deficit. However, knowledge gaps remain, including the optimal dose of EAA during the catabolic stress of energy deficit, as well as how whole-food-containing mixed meals directly compare to supplemental free-form EAA and isolated intact proteins. Lastly, future studies should include simultaneous assessments of MPS, muscle protein breakdown, and whole-body protein balance to fully evaluate the efficacy of feeding formats in supporting body protein balance. Systematically addressing these gaps, while clarifying previous work, will provide the information required to quantify the relationship between feeding format and MPS as well as whole-body protein balance. This will ultimately aid in practical recommendations for when supplemental free-form EAA, supplemental intact protein, and protein-containing mixed meals should be consumed to optimally support total-body protein during energy deficit.
